# Supplementary material for: Accelerating joint species distribution modelling with Hmsc-HPC by GPU porting
Source: PLoS Comput Biol. 2024 Sep 3;20(9):e1011914. doi: 10.1371/journal.pcbi.1011914 (PMC11398642; doi:10.1371/journal.pcbi.1011914)
Supplement: Attachment — Submitted filename: rebuttal.pdf [file pcbi.1011914.s001.pdf]

Original Manuscript ID: PCOMPBIOL-D-24-00267

Original Article Title: Accelerating joint species distribution modeling with Hmsc-HPC: A 1000x faster GPU deployment

Revised Article Title: Accelerating joint species distribution modelling with Hmsc-HPC by GPU porting

To: PLOS Computational Biology

Re: Response to reviewer comments

Dear Editor,

Sincerest thanks for your response and reviewer comments on our manuscript. We have modified the paper in response to the extensive and insightful reviewer comments.

We have added more information to address the reviewers' comments fully. Consequently, we hope the revised manuscript alongside the responses complies with all the remarks.

Herewith, we are uploading (a) a point-wise response to reviewers, (b) an annotated manuscript with revisions highlighted as coloured text, and (c) the revised manuscript.

Sincerely,

Anis Ur Rahman

Rebuttal

Manuscript # PCOMPBIOL-D-24-00267

Accelerating joint species distribution modelling with Hmsc-HPC by GPU porting

This document provides a statement of the revisions to manuscript # PCOMPBIOL-D-24-00267: "Accelerating joint species distribution modelling with Hmsc-HPC by GPU porting." We would like to thank the reviewers for their valuable comments, which facilitated us to improve our manuscript. We considered each comment and made the necessary modifications in the revised version. Our revisions reflect all the reviewer's suggestions and comments. The revised version of the paper, based on the review team's collective input, includes several improvements to the original submission.

In this document, we added a point-by-point reply to the reviewers' recommendations. To improve the readability of this document, and to help differentiate the reviewer's comments from our response, we have used different colour formatting in this document.

Black colour: Represents the reviewer's comments.

Blue colour: Represents the authors' response to the reviewer.

Table of Responses to Reviewer Comments

Reviewer #	Reviewer Issue/Comment to Be Addressed	Response/Action Taken	Document Location
1	Rahman et al. present an exciting GPU implementation of Hmsc to fit a multitude of complex joint species distribution models. This is a very impressive contribution, and one that will be utilized greatly by the ecological community. Overall, I found the manuscript an appropriate length for communicating a software advancement. However, I found certain aspects of the manuscript to potentially overstate the value of Hmsc-HPC, particularly in the context of fitting spatially-explicit models, which I believe the authors should address prior to publication.	Thank you for your valuable comments and feedback. Your comments have greatly helped improve the quality of our submission.	-

1	Major comments<br><br>1. Benefits of Hmsc-HPC for spatial models: Figure 2 clearly highlights the benefits of Hmsc-HPC for non-spatial and phylogenetic models, which is quite exciting given these models likely comprise a large majority of the models fit by the Hmsc user community. However, as the authors point out, there were minimal benefits, if any at all, for the GPU implementation of NNGP models, which also showed fairly close correspondence with the model run times for the GP and predictive process models when implemented in Hmsc-HPC. This leads me to ask the question: is it worthwhile for an Hmsc user who wants to fit a large spatial model to use the GPU implementation over the existing Hmsc-R package functionality for NNGPs?	Thank you for raising this important question. The benefits of our GPU implementation for NNGP models compared to non-spatial and phylogenetic models are indeed minimal.<br><br>The reasons for the little benefit of our GPU implementation for NNGP are briefly explained at the end of the "Predictive performance" section. In somewhat more detail, the algorithm implemented in Hmsc-R, also adapted in Hmsc-HPC, is based on the "collapsed model" approach for sampling NNGP, as outlined in Finley et al. (2019). This method relies on sparse Cholesky factorisation, chosen for its superior MCMC mixing properties compared to the "sequential model" proposed by Datta et al. (2016).<br><br>While most of the operations used by Hmsc-HPC's sampling algorithm are available in TensorFlow, the sparse factorisation routine is not. To the best of our knowledge, its sequential nature makes it unsuited for GPU parallelization. Thus, our NNGP implementation relies on CPU-based calls to `scipy` routines.<br><br>However, executing part of the algorithm on the CPU introduces overhead due to massive CPU-GPU communication, a problem absent in other Hmsc-HPC models. Accelerating NNGP models in Hmsc-HPC would necessitate a GPU-friendly sparse Cholesky decomposition implementation. Yet, given the limitations of existing algorithms, we are sceptical about its feasibility in the near future.<br><br>While NNGP remains the default recommendation for large spatial data in Hmsc-R, it is not considered the pinnacle of existing approximations for spatial GP. Recent successors to NNGP offer superior computational properties. Some of these methods, like block-NNGP by Quiroz et al. (2023), are well-suited for GPU porting, replacing observation-wise neighbour dependence with block-wise dependence. Similarly, Peruzzi et al. (2022) propose meshed GP, which operates on subsets of observations jointly, reducing the issue of slow MCMC mixing in the "sequential	See p. 7, L282-295<br><br>See p. 8, L318-324

		model." We plan to implement the block-NNGP approach in the upcoming expansion to the Hmsc-HPC. However, as we elaborate below, already the current Hmsc-HPC implementation provides substantial benefits for users fitting spatial models, as compared to applying NNGP with the baseline R-package Hmsc.<br><br>We have provided a more detailed discussion on this topic in the revised version of our manuscript, where we better linked the problem to published research, and outline the prominent direction of further improvement.<br><br>References:<br><br>Finley, Andrew O., et al. "Efficient algorithms for Bayesian nearest neighbor Gaussian processes." Journal of Computational and Graphical Statistics 28.2 (2019): 401-414.<br><br>Datta, A., Banerjee, S., Finley, A. O., & Gelfand, A. E. (2016). Hierarchical nearest-neighbor Gaussian process models for large geostatistical datasets. Journal of the American Statistical Association, 111(514), 800-812.<br><br>Quiroz, Z.C., Prates, M.O., Dey, D.K. et al. (2023) Fast Bayesian inference of block Nearest Neighbor Gaussian models for large data. Stat Comput 33, 54.<br><br>M Peruzzi, S Banerjee, AO Finley (2022). Highly scalable Bayesian geostatistical modeling via meshed Gaussian processes on partitioned domains. Journal of the American Statistical Association 117 (538), 969-982.	
1	It is clear from Panel B that Hmsc-HPC yields clear benefits for fitting full GP models, but given the very good performance of NNGP models at closely approximating a full GP (e.g., Datta et al. 2016), is it realistic to expect a GP JSDM to be fit by an ecologist with anything more than 100-200 spatial locations?	In our computational performance experiment, we fitted a full GP model with 10 latent factors to data from 1600 distinct spatial sites. As indicated in Fig 2F, the runtime for 200,000 MCMC iterations was around 1 day with Hmsc-HPC, compared to an estimated 5 years with Hmsc-R for an identical model fit. For a full GP model, computational load scales as (ny*nf)^3 in FLOPS and (ny*nf)^2 in allocated memory (ny is the number of sites and nf is the number of latent factors). Our findings	

		confirm that Hmsc-HPC realistically handles spatial datasets containing up to around 1000 spatial locations, given the number of factors remains relatively small, without requiring spatial GP approximation. However, the effective number of factors is study-specific and can be as large as the number of modelled species. Yet, many studies report a low estimated number of factors.<br><br>The practical utility of Hmsc-HPC is supported by the fact that fitting data with 1600 spatial units and 622 species: NNGP variant in Hmsc-R on CPU takes 58 hours, whereas the full GP variant in Hmsc-HPC on GPU takes 23 hours. Thus, while ecologists may struggle to fit full-GP JSDMs with CPU-bound software, our GPU-accelerated framework provides a solution, enabling the analysis of datasets with thousands of spatial locations.	
1	Further, while the predictive process model also showed substantial improvements in Hmsc-HPC, predictive process models and other low rank approximations to GPs are known to oversmooth (Stein 2014), which is something many of the authors of this manuscript found in a previous manuscript with this exact data set (Tikhonov et al. 2020). NNGPs do not have the same oversmoothing issues. While the predictive process models with Hmsc-HPC did have lower run times across the board compared to the NNGP models in Hmsc-R, it is not possible to directly compare the approaches due to (1) potential differences in mixing/convergence of the MCMC chains; (2) differences in model performance that may result from using too few knots in the predictive process approximation (here the authors use 55 while in Tikhonov et al. 2020 1026 knots were used, a substantial increase in run time) or too few neighbors (10	Thank you for your detailed comment and for referencing the earlier work by Tikhonov et al. (2020). We appreciate your insight into the potential differences between the predictive process models and the NNGP models for the key parameters of these approximations that affect both the computational load and model fit quality, e.g. in terms of predictive performance. In our current study, our primary focus was on evaluating the computational performance of Hmsc-HPC relative to Hmsc-R, particularly in terms of runtime efficiency. As such, we chose to use identical approximation parameters between the competing Hmsc-R and Hmsc-HPC to facilitate a direct comparison of computational performance. Simultaneously, we tried to avoid conducting any elaborate assessments of which approximation is better per se, as such work has been conducted before and would disperse the focus of our current study.<br><br>As we have mentioned above, we support your statement that for making informed recommendations for ecologists of what method to use for fitting spatial JSDMs, a more comprehensive comparison study would be required. Ideally, such a study would assess multiple aspects of existing software using multiple real datasets	See p. 7, L291-295<br><br>See p. 8, L319-324

	neighbors here vs. 20 in Tikhonov et al. 2020). I know this is not the objective of the performance comparison, as the authors point out in lines 210-214. However, given the lack of substantial improvements for the NNGP models, and the potential for the NNGP models implemented in Hmsc-R to outperform predictive process models implemented in Hmsc-HPC, I do not feel that there is conclusive evidence of Hmsc-HPC providing improvements in fitting spatial models relative to Hmsc-R. In my opinion, this would require a substantial comparison study of the models in terms of run times to convergence, predictive performance, etc. This would be a substantial undertaking, and something I do not think is necessary for this manuscript. I am not denying the benefits of Hmsc-HPC for fitting nonspatial or phylogenetic models; those findings are extremely clear. However, I believe the authors should more explicitly state that a more robust assessment/comparison of the NNGP/predictive process models under both platforms should be a future avenue of research in order to best determine the most effective approach to fitting spatial models.	covering a broad range of taxa and spatial scales. Clearly, assessment of predictive performance and computational load would be the criteria of primary interest, but other characteristics of the compared software shall be considered.<br><br>To address your comment, we explicitly state the need for a robust assessment and comparison of the NNGP and predictive process models under both platforms as a future avenue of research in our manuscript.	
1	2. Title: the "1000x faster GPU deployment" is context dependent, as it depends on the model type and size of the data set. The 1000x improvement, as the authors point out in the abstract, is for the largest data set (over 20,000 points), which I would guess is larger than the data sets most Hmsc users work with. Thus, I find this title overstated, and would suggest removing the "1000x faster GPU deployment" and instead	We appreciate your perspective on the context-dependent nature of the "1000x faster GPU deployment" claim and agree that it is essential to provide accurate and contextually appropriate information to our readers. Given your feedback, we have revised the title as “Accelerating joint species distribution modeling with Hmsc-HPC by GPU porting”. We now mention in the abstract that “Notably, for the largest datasets, we achieved >1000 times speed-ups”.	

	only including that in the abstract where the proper context for it can be provided.		
1	Minor comments:<br><br>+ Line 26-28: it may not be clear to ecologists reading the paper that "Hmsc-HPC" is not a new R package, and rather is an extension written in Python that needs to be installed separately from the Hmsc-R CRAN implementation. I would suggest including this information explicitly here.	Thank you for highlighting the potential confusion regarding "Hmsc-HPC" as an extension written in Python rather than a standalone R package. We have revised the manuscript to explicitly clarify this distinction, ensuring that readers understand the nature of Hmsc-HPC as an extension of the Hmsc-R package.	See p. 3, L94-96
1	+ Line 137-138: does this mean the user must have familiarity with Python, or is this all accomplished directly through R? Some more detail, and potentially a reference to an online example of how this is done with the package, would be helpful.	No prior Python coding experience is necessary for utilizing Hmsc-HPC. However, installation of the extension is required. We have revised our accompanying notebook (available online at link) by including an overview of the typical Python installation process and links to dedicated guides. This shall ensure accessibility for users regardless of their Python proficiency level. Thank you for bringing this to our attention.	
1	+ Lines 192-195: the authors do not give appropriate citation for the predictive process (Banerjee et al. 2008) or NNGPs (Datta et al. 2016). These should be cited accordingly.	Corrected.	
1	References<br><br>+ Banerjee, S., Gelfand, A. E., Finley, A. O., & Sang, H. (2008). Gaussian predictive process models for large spatial data sets. Journal of the Royal Statistical Society Series B: Statistical Methodology, 70(4), 825-848.		

	+ Datta, A., Banerjee, S., Finley, A. O., & Gelfand, A. E. (2016). Hierarchical nearest-neighbor Gaussian process models for large geostatistical datasets. Journal of the American Statistical Association, 111(514), 800-812.<br><br>+ Stein, M. L. (2014). Limitations on low rank approximations for covariance matrices of spatial data. Spatial Statistics, 8, 1-19.<br><br>+ Tikhonov, G., Duan, L., Abrego, N., Newell, G., White, M., Dunson, D., & Ovaskainen, O. (2020). Computationally efficient joint species distribution modeling of big spatial data. Ecology, 101(2), e02929.		
2	I revised the manuscript entitled “Accelerating joint species distribution modeling with Hmsc-HPC: A 1000x faster GPU deployment”. In this manuscript, authors introduced a new package to create JSDM. The manuscript is straightforward and clear, and the experiment performed to test the performance between Hmsc-R and Hmsc-HPC is appropriate. The tutorial provided also worked well and was very didactic. Perhaps, in the future, the package's authors could create a web page (e.g., with pkgdown) with tutorials for different modeling situations. One of the points I would like to highlight about the authors is that they integrate the two programming languages (R and Python). This will undoubtedly stimulate the use of Hmsc-HPC by former Hmsc-R users and others who only use R as a programming language. It is very exciting to see the speed at which JSDMs can be	Thank you for your thorough and positive feedback on our manuscript. We appreciate your recognition of the clarity of our manuscript, the appropriateness of our performance experiment, and the effectiveness of the tutorial provided. Your suggestion to create a web page with tutorials for different modelling situations using pkgdown is duly noted and will be considered in the future release of the package. We also acknowledge your appreciation for our integration of R and Python and share your excitement about the potential impact of our package on the development and use of joint species distribution models.	

	constructed, which in many other conditions would be prohibitive. I believe that the improvements that this package will bring to the already widely used Hmsc-R package will be of extreme importance for the development of these models, enhancing their use in new applications in regions with many samples or with many species.		
3	The manuscript presents a new GPU-based, implementation of the HMSC fitting software for Joint Species Distribution Models. HMSC fits JSDMs in a Bayesian setting using MCMC, but since the original package implements those routines in the R programming language a large speed-up is to be gained by using the TensorFlow library in python for model fitting instead. This potentially greatly facilitates fitting JSDMs with complex model structures to large datasets in the Hmsc software.	Thank you for this positive evaluation of our work.	
3	Although the manuscript is generally well written, it contains some typos that I have attempted to outline in the detailed comments, but I generally recommend the authors to thoroughly re-read their text for potential errors that I (or another reviewer) might have missed. Generally, I find many sentences that are much less succinct than possible, and combined with the excessive use of unnecessary adjectives, the text at times feels convoluted and lacks the flow that makes for an enjoyable read.	We have carefully reviewed the entire manuscript in light of your feedback and have made numerous necessary modifications to streamline the writing, eliminate unnecessary adjectives, and improve the overall flow of the text to enhance readability.	Entire manuscript

3	I applaud and thank the authors for including a reproducible script of their analyses, and installation instructions for their new software package, this made trying out their implementation easier, generally more enjoyable, and trying the implementation clarified various aspects of the manuscript for me.	We're glad to hear that trying out our implementation enhanced your understanding of the manuscript. Your appreciation is greatly valued.	
3	The selected literature in the first paragraphs is somewhat narrow, and although the article makes some mention of alternative (fast) software for JSDM fitting, it ignores large parts of the literature that were developed in recent years for fitting JSDMs. This includes the glmmTMB R-package, or the gllvm R-package whose main developer is of course at the same university as the last author of the current manuscript, but also e.g., copula-based methods (Popovic et all. (2022)), quasi-likelihod based methods (Kidzinski et al. (2022)), or the basis-function approach from Hui et al. (2023). Either how, Pichler and Hartig (2021) as a reference on L25 seems inappropriate as that reference does not speak to the "hinders of scientific discovery" due to computational limitations, but suggests a different method for fitting JSDMs based on an elastic net penalty.	We acknowledge the oversight in the original manuscript regarding the recent introduction of scalable software for fitting JSDMs. Our original focus was intentionally limited to the published methods with existing GPU implementations. In response to your feedback, we have expanded the introduction to include a paragraph providing an overview of published methods aimed at scalability in JSDM fitting. We outline their key concepts, highlight differences between approaches and explain why Hmsc-R remains relevant for practitioners despite being computationally less attractive than some of the alternatives.	See p. 2-3, L54-82
3	There are various general statements on the benefit that this computational improvement is supposed to have on biodiversity research, e.g., "far-reaching implications for informing conservation strategies" or "a profound advancement in the	Thank you for your feedback regarding the language used to describe the computational improvement and the perceived novelty of our GPU implementation in biodiversity research. We appreciate your perspective and	Entire manuscript

	domain of community ecology". Although those might be true, since there is no comparison with other fitting methods (e.g., with the software by Pichler and Hartig (2021) that the authors cite), the reader is left wondering if this present GPU implementation is only a speed-up over the existing Hmsc software, or truly an advance for fitting JSDMs in all of community ecology as the manuscript states. Without comparison with other software implementations, it is not possible to assess the latter, and the manuscript feels like it is overselling its novelty, as it pertains a quite limited internal comparison based on a single dataset. My suggestion is that the authors tone down the language, or alternatively include a comparison with other software packages. The chosen title has a similar issue, since in the manuscript it becomes clear that there is not always a 1000 times speed-up, but only compared to the regular Hmsc implementation, and only for the single dataset with which a computational comparison has been performed.	agree that it's essential to provide a balanced representation of our contribution.<br><br>In light of your comments, we acknowledge that our GPU implementation primarily serves as a speed-up over the existing Hmsc software, rather more generally advancing also other JSDMs. We scrutinously revised the writing and toned down the used language and claims made. Specifically, we rephrased the sentences that previously potentially implied that our implementation addresses all existing JSDMs.	
3	Generally, I would appreciate some disclaimer in the text when it comes to user friendliness, as most ecologists fitting JSDMs will not be familiar with python (though it seems only a limited degree is necessary for use of HMSC-HPC), and performing a python installation, or debugging it, can be very challenging (and the vignette provided by the authors essentially states to google a problem if a user cannot figure it out, offering only very limited support even to the	We fully agree that user-friendliness is a cornerstone for an ecology-oriented R package in order to become widely used. Indeed, potential challenges related to Python installation can impose a steeper learning barrier for ecologists who are often unfamiliar with the language. Given our focus on practitioners, we prioritise providing clear guidance and support, especially for those with limited Python experience.<br><br>To address this, we have included an overview of the typical Python installation process in the revised notebook (available	See p. 2, L35-37

	reviewers..fortunately I did not need any!).	online at link), ensuring accessibility regardless of proficiency level. Unfortunately, due to potential variations in hardware, OS, and user rights, producing a comprehensive set of uniformly applicable guidelines is close to infeasible. For example, configuring TensorFlow for NVIDIA cards on a university-owned Windows machine posed a fair amount of challenges for us (and university IT support) as well.<br><br>Simultaneously, we highlight that the design of the proposed package makes no assumptions about any other competency in Python beyond its installation. Neither coding skills nor familiarity with debugging of Python code (or TensorFlow graphs) is required or expected from a regular user.	
3	Out of curiosity, why did the authors decide to develop an add-on package, while keeping R as the default backend for HMSC, if use of the TensorFlow library has so much benefits?	Full transition of an analysis pipeline from R to Python would be challenging for ecologists with limited Python experience. Furthermore, many commonly used preprocessing and postprocessing steps often rely on R-only packages that are not readily available in Python.<br><br>Developing Hmsc-HPC as an add-on package allows users to opt for high-performance computing (HPC) with the understanding of additional costs and learning requirements. Retaining the R interface ensures accessibility for ecologists familiar with R workflows.<br><br>While there is a TensorFlow package for R, it relies on `reticulate` package and requires Python installation, offering no advantage to reducing the Python configuration requirement. Additionally, it is vastly less documented than the original TensorFlow package for Python language. Taking these factors into account, at the initial stage of this research project we made a strategic decision to choose Python+TensorFlow for our add-on development.	

3	Finally, I find the comparison based on a single dataset, and without any other software packages than that of the authors, very limiting. The authors even use a proxy for fitting Phylogenetic models as the dataset that they perform comparisons on does not actually seem to include Phylogenetic information. I advice the authors to expand on their comparison, use one or two more datasets, or just perform a complete set of simulations based on (a select few of) the various model structures that the Hmsc package is capable of fitting.	We wholeheartedly support your proposal that a comparison of our implementation against other published methods using multiple datasets would greatly benefit the comprehension of the rapidly expanding landscape of existing JSDM implementations. In our opinion, such a comparison could be designed alike the work of Norberg et al (2019) in the sense that it would primarily focus on the performance assessment of realistic data analysis tasks with several diverse real data cases. But unlike this past work published 5 years ago, a thoughtful modern comparison shall be expanded to assess the recently published methods, as well as additionally take into account computational times and big data scalability aspects.<br><br>In our view, such a comparison work is far beyond the scope of our implementation-targeted manuscript. We have now clarified that the primary focus of our study is on computational performance rather than on predictive performance. Given that we do not introduce any changes (in terms of mathematical logic) to the model fitting algorithm compared to the R-package Hmsc, in our view, it is sufficient to only evaluate the ratio of elapsed time per MCMC iteration. Moreover, such elapsed time per MCMC iteration is dependent on the selected model structure and data dimensions only, but not on the very data itself. Therefore, we consider that the provided timings for 5 commonly used distinct HMSC types: GLLVM, GLLVM with phylogeny information, and 3 variants of GLLVM + spatial dependencies, with varied numbers of sampling units and number of species, already provide a comprehensive set of reference points for an interested Hmsc user to assess the benefit of switching to the HPC version. In the same line, whether one uses a quantitative phylogeny or taxonomically-induced phylogeny does not change the computational speed up, and	

		hence we consider our example sufficient also from this point of view.<br><br>References:<br><br>Norberg et al. A comprehensive evaluation of predictive performance of 33 species distribution models at species and community levels. Ecological Monographs. 2019	
3	Detailed comments<br><br>Please number the manuscript throughout. The abstract and author summary were left unnumbered, leaving reviewers to manually count lines to provide comments, which is clearly not impossible, but unnecessary tedious.	Corrected.	
3	Abstract<br><br>L3: “However, JSDM...” → “However, fitting or estimating JSDMs” or similar. Also, clarify what is meant here by “prohibitive”. I suppose you mean that the long computation time is prohibitive, but JSDMs in and of itself are not.	Here's a revised text is:<br><br>"However, fitting JSDMs to large datasets is often computationally demanding and time-consuming. Recent studies have introduced new statistical and machine learning techniques to provide more scalable fitting algorithms, but extending these to complex JSDM structures that account for spatial dependencies or multi-level sampling designs remains challenging."<br><br>This revision clarifies that it is the computational aspect of fitting or estimating JSDMs that can be prohibitive, particularly of those JSDM that attempt to take into account auxiliary data (such as phylogeny) due to long computation times, especially with large datasets and complex model structures.	See p. 1, L4-9

3	L6: Please rephrase. Your GPU implementation does not address computational limits of JSDMs generally, purely as implemented in the Hmsc software.	Corrected. Also, this is made explicit in other ambiguous sentences throughout the manuscript.	See p. 1, L6-9
3	L9: "TensorFlow" → "the TensorFlow"	Corrected.	
3	L10: "primarily targets to enable" → "enables"	Corrected.	
3	L12: Please remove repeated statement from L10	Corrected.	
3	L17: "boosts the scalability of Hmsc-R package" → "boosts scalability of the Hmsc R-package" and remove "reaching a significantly higher level" in the second part of the sentence	Corrected.	
3	L19-L20: I will get back in my major comments about statements like these throughout the manuscript, but generally I think this is an overstatement w.r.t. the benefit of the improved method. It is great that models in Hmsc can now be fitted much faster, but there are other (potentially) faster algorithms that fit similar/the same JSDMs available, so to state that this particular GPU implementation is going to enable better informed conservation strategies, environmental management, and climate change	We agree and have been more specific in our revision about the scope of our work. The revision acknowledges the potential benefits of the GPU implementation while also recognizing the existence of other potentially even faster algorithms for fitting similar JSDMs. It refrains from making overly bold claims about the specific impact of our implementation on conservation strategies, environmental management, and climate change adaptation planning.	Entire manuscript

	adaptation planning, seems very unlikely.		
3	Author summary<br><br>I somewhat take issue with the statement that the authors are "retaining the user-friendly R interface", while later on L137-147 it becomes clear that the user actually needs to perform operations in an independent python session (albeit from R). I would like the authors to be more transparent on how their implementation requires interfacing independent use of R and python instead, as that is not a minor feat for many ecologists.	Here's a revised version that clarifies the interface between R and Python:<br><br>"Our updated framework maintains the original ecologist-friendly R interface while integrating Python and TensorFlow into its computational core. This integration provides seamless access to GPU computing, thereby improving the efficiency of high-performance computing resources. Users need to set up Python to execute the model fitting, but no coding skills are required to benefit from GPU acceleration."<br><br>This revision aims to provide a more transparent explanation of the interface between R and Python, addressing the concerns raised by the reviewer.	See p. 2, L32-37
3	Introduction<br><br>L8: JSDMs were introduced >10 years ago, I am not sure I agree with the authors that I would call this "recent"	Corrected.	
3	L13: The reference (4) cited in this sentence includes a comparison of predictive performance for JSDM fitting softwares and has little to do with fitting algorithms, which is what that this sentence is currently about. Please either 1) cite software packages or different fitting algorithms here instead, or 2) alter the sentence to target predictive performance as that is what this reference studies. Additionally, the reference is five years old and there	We reworked the sentence. Now we are citing multiple references to papers that introduced readily reusable JSDM software.	See p. 2, L54-69

	have been many computational developments in fitting JSDMs that it does not cover.		
3	L14: Difficult to read sentence, suggest rewriting to "HMSC facilitates researchers to study the environmental niche of species, potentially structured by additional traits and phylogenetic information" or similar.	Corrected.	
3	L16: remove "regression-style" as it does not add anything to the sentence	Corrected.	
3	L19: Again, I suggest changing "allows" to "facilitates" since researchers were also allowed to fit JSDMs before HMSC was developed.	Corrected.	
3	L20: "post-process for prediction and inference" rarely can model results be processed for prediction and inference, since models are (should) usually be calibrated for one or the other (I have yet to meet a model that is suitable for prediction and inference). So I suggest the authors change this statement to "prediction or inference".	Thank you for your feedback regarding the terminology used in our manuscript. While we appreciate your suggestion to change the statement to "prediction or inference," we respectfully disagree. In our view, in many modeling contexts, the same models are calibrated to perform both prediction and inference simultaneously.<br><br>Prediction refers to the model's ability to make predictions on new, unseen data, while inference involves drawing conclusions about the underlying relationships between variables based on the fitted model and observed data.<br><br>Many models are designed to serve both purposes simultaneously. For example, in machine learning, models like decision trees, random forests, and neural networks are	

		trained to make predictions on new data while also providing insights into the relationships between variables, which is a form of inference.<br><br>We believe our original statement accurately reflects the dual purpose of post-processing model results for both prediction and inference. However, we understand the importance of clarity and will ensure that the terminology used in our manuscript accurately reflects the intended meaning.	
3	L20: “Despite its utility” – please elaborate, what utility, or utility of what tool, do the authors refer here to?	Corrected.	
3	L23-24: the models in HMSC can be fitted with different software, so I doubt that this statement is true. The availability of HMSC makes that these models can be fitted in a more user friendly setting, and by researchers with less knowledge of model fitting algorithms, so that it makes for generally more robust inference.	We have toned down this sentence.	
3	L29: “Optimizing” implies that the existing model fitting process is made more efficient, which I am not sure is the case here, because the GPU implementation actually facilitates the use of a different set of computational resources that were not previously available to the Hmsc-R user.	We appreciate your attention to detail regarding the use of the term "optimizing" in the manuscript. While the GPU implementation does indeed introduce a different set of computational resources, our intention with the term "optimizing" is to emphasize the enhancement of the efficiency of the model fitting process. By transitioning to a TensorFlow-based computational backend with GPU acceleration, we aimed to streamline the model fitting process and improve its efficiency compared to the	See p. 3, L96-99

		previous R-based calculations. We hope this clarification addresses your concern.	
3	L37-L42: This statement may be true, although to be honest I doubt that it is. The authors do not provide an exhaustive comparison with other JSDM fitting softwares, so there is no way to verify that the software that the authors have developed actually fits these models faster than any other software currently available. And that would be a requirement for "a profound advancement in the domain of community ecology…".	Thank you for your insightful comments regarding the statement on the advancement in the domain of community ecology. We have revised the corresponding statement in the manuscript, discarding the overstatement of its profound impact. The revised statement now reads: "By harnessing the parallel processing capabilities of GPUs, we significantly expedite the execution of the block-Gibbs sampler used in HMSC fitting. This acceleration markedly reduces the time required for model fitting and alleviates the performance bottleneck associated with input size, thereby enhancing the package's ability to handle extensive datasets that were previously computationally prohibitive."<br><br>As we have mentioned above, we acknowledge your point regarding the need for an exhaustive comparison of existing JSDM fitting software to assess the effectiveness of our technical enhancement fully. This verification will be an essential aspect of future research endeavours, ensuring that contributions of existing JSDM implementations are rigorously evaluated and validated within the broader context of existing methodologies. Thank you for bringing this important consideration to our attention.	See p. 3, L99-103
3	L47: "intricate" is probably not how most ecologists would describe their study designs. Perhaps "nested" or "complex" is more appropriate here.	Corrected.	

3	L49: “Generalised regression” is not a method I am familiar with. Do the authors refer here to the GLM-framework?	Thank you for pointing out this potential confusion. Indeed, we intended to refer to the GLM class of models.	
3	L50: “resulted” → “resulting”	Corrected.	
3	L51 “in Bayesian paradigm” → “in the Bayesian paradigm”	Corrected.	
3	L52: MCMC does not make for a more versatile modeling framework than via other fitting algorithms, so please rephrase this statement.	Corrected.	
3	L55: “Prediction and inference” → “Prediction or inference”, see comment w.r.t L20 where this was also stated in the same manner.	See our response above.	
3	Fig 1. … “with Hmsc-R package” → “with the Hmsc-R package”. Also, throughout, please rephrase “Hmsc-R package” to “Hmsc R-package”.	Corrected.	
3	Unfortunately, I could only access the figures in very low resolution as attached to the manuscript, so that they were near impossible to assess.	We are sorry about this. We have submitted high-quality versions of the figures separately; however, they are appended to the manuscript as pdfs with much lower quality by the journal submission system. You should find links to the original high quality versions on top right corner of the pages with the figures.	

3	L75: "Inference and predictions" → "Inference or predictions"	See our response above.	
3	L81: remove "prohibitive" as it does not add anything to this sentence.	Corrected.	
3	L82-85: you might want to draw comparison to approximate Bayesian methods for fitting JSDMs instead of MLE here, such as Inla or empirical Bayes methods.	Corrected.	See p. 4, L139-145
3	L88-91: This is an excellent sentence!	Thank you.	
3	L93-94: "...explore what extent it can be … hardware." → "explore to what extent it can be enhanced, when substituting the original model fitting implementation with an algorithm suited for HPC hardware" or similar.	Corrected.	
3	Many of the sentences in the manuscript can be made more succinct without losing information, making the whole manuscript more enjoyable to read.	Thank you for your feedback; we have revised the manuscript to make the sentences more succinct and enhance readability without losing essential information.	
3	L96: This sentence makes me wonder if I can use your new implementation for parallel computing with CPU instead, can the authors add something on that here? (I later read a statement on this on L143, but the point remains that the statement should be moved up in the text)	Yes, TensorFlow, the underlying framework used in our implementation, indeed offers support for executing operations across multiple threads and exploiting parallelism, enabling efficient utilization of both GPU and CPU resources. While our main focus is to leverage GPU resources available on HPC systems, which drives several design decisions in the implemented TensorFlow	See p. 4, L173-175

		code, TensorFlow's flexibility allows for effective utilization of CPU resources as well. We have revised the manuscript to include this information earlier.	
3	L101: remove “numerous” since this really depends on the machine/cluster that is being utilized	Corrected.	
3	L102: “decomposes” is perhaps not the right word here. A more appropriate phrasing might be “Specifically, the bulk of operations within the block-Gibbs algorithm consists are algebraic in nature, so that they can be formulated within…”	Corrected.	
3	L103: “These computations can be parallelized” → “The computations can then be parallelized”	Corrected.	
3	L104: remove “multiple” as it is already implied. Also remove “the” before “simultaneous”	Corrected.	
3	L107-108: rephrase to something like “We redesigned and re-implemented the Hmsc-R block-Gibbs sampler routine in the Python language using the …”	Corrected.	
3	L127: “with TensorFlow-based” → “with the TensorFlow-based”	Corrected.	

3	L154: remove "numerous" again	Corrected.	
3	L188: "experimental design" → "design". The experimental is implied by the first "experiments"	Corrected.	
3	L199-201: this seems a bit odd, why do the authors not just introduce a second example dataset that actually has a Phylogeny available with it?	We appreciate your suggestion to introduce a second example dataset that includes explicit phylogenetic information. While we understand the value of such a dataset for a comprehensive evaluation of phylogenetic models, we want to emphasize that our primary focus in this study is on computational performance rather than predictive performance. Therefore, we opted to use a proxy for fitting phylogenetic models, even if the dataset does not contain explicit phylogenetic information. This approach allows us to accurately assess the running time performance of different model structures and implementations within the scope of our study. We furthermore note that often users of Hmsc do not have quantitative phylogenies available and that for such cases using a taxonomy-based tree is often the best available solution. However, we acknowledge the potential benefit of including a dataset with explicit phylogenetic information in future research to provide a more comprehensive evaluation.	See p. 8, L332-336
3	L224-237: I would be happy for further clarification here. Although (apparently) parallelized GPU computation is not possible for the models that use a NNGP, parallel CPU computations are still available? No speed-up compared to Hmsc routines implemented in R seems very surprising, especially as e.g., Finley et al. (2019) conclude that NNGP	We appreciate your thorough analysis and the points you've raised regarding the potential for parallel computing with the collapsed NNGP models. However, we would like to refer to the pseudocode of the collapsed NNGP model (as presented by Finley et al. (2019)), which is the closest equivalent of its implementation in HMSC. It's important to note that while some parts of the computations e.g. the loops in the	See p. 7, L282-295<br><br>See p. 8, L319-324

	approximations are highly parallelizable.	pseudocode, can indeed be parallelized, there are key operations that operate with sparse matrices, such as the sparse Cholesky decomposition:<br><br>(d)L=sparsechol(sparsemm(sparsemm(P,Ω),P^T)),<br><br>This operation remains computationally intensive and is not effectively parallelized, even in a CPU setting. As indicated in Figure 2a of Finley et al. (2019), the authors found no benefit of using multiple CPU cores for the collapsed model. Furthermore, we cite the Summary section of Finley et al. (2019) on this regard:<br><br>"So while the Collapsed model can be computationally intensive, depending on the burden imposed by the sparse Cholesky decomposition, it is the only fully Bayesian alternative to the sequential Gibbs sampler developed in Datta et al. (2016a) and should generally be selected over the latter due to its significantly improved chain convergence."<br><br>Thank you for bringing attention to these important considerations, which underscore the complexities involved in parallelizing computations for collapsed NNGP models.	
3	References<br><br>Finley, Andrew O., et al. "Efficient algorithms for Bayesian nearest neighbor Gaussian processes." Journal of Computational and Graphical Statistics 28.2 (2019): 401-414.<br><br>Hui, Francis KC, et al. "Spatiotemporal joint species distribution modelling: A basis function approach." Methods in Ecology and Evolution 14.8 (2023): 2150-2164.		

	Kidzinski, L., Hui, F. K., Warton, D. I., & Hastie, T. J. (2022). Generalized Matrix Factorization: efficient algorithms for fitting generalized linear latent variable models to large data arrays. Journal of Machine Learning Research, 23(291), 1-29.<br><br>Niku, J., Brooks, W., Herliansyah, R., Hui, F. K. C., Korhonen, P., Taskinen, S., van der Veen, B., and Warton, D. I. (2024). gllvm: Generalized Linear Latent Variable Models. R package version 1.4.5.<br><br>Popovic, Gordana C., Francis KC Hui, and David I. Warton. "Fast model-based ordination with copulas." Methods in Ecology and Evolution 13.1 (2022): 194-202.		
